# Frequency dependent emotion differentiation and directional coupling in amygdala, orbitofrontal and medial prefrontal cortex network with intracranial recordings

**DOI:** 10.1038/s41380-022-01883-2

**Published:** 2022-12-02

**Authors:** Saurabh Sonkusare, Ding Qiong, Yijie Zhao, Wei Liu, Ruoqi Yang, Alekhya Mandali, Luis Manssuer, Chencheng Zhang, Chunyan Cao, Bomin Sun, Shikun Zhan, Valerie Voon

**Affiliations:** 1 Department of Neurosurgery, Centre for Functional Neurosurgery, Ruijin Hospital, Shanghai Jiao Tong University School of Medicine, Shanghai, China; 2 Department of Psychiatry, University of Cambridge, Cambridge, UK; 3 Institute of Science and Technology for Brain-Inspired Intelligence, Fudan University, Shanghai, China; 4 Key Laboratory of Computational Neuroscience and Brain-Inspired Intelligence, Fudan University, Ministry of Education, Shanghai, China; 5 Nuffield Department of Clinical Neurosciences, University of Oxford, Oxford, UK; 6 MRC Brain Network Dynamics Unit, University of Oxford, Oxford, UK

**Keywords:** Neuroscience, Physiology

## Abstract

The amygdala, orbitofrontal cortex (OFC) and medial prefrontal cortex (mPFC) form a crucial part of the emotion circuit, yet their emotion induced responses and interactions have been poorly investigated with direct intracranial recordings. Such high-fidelity signals can uncover precise spectral dynamics and frequency differences in valence processing allowing novel insights on neuromodulation. Here, leveraging the unique spatio-temporal advantages of intracranial electroencephalography (iEEG) from a cohort of 35 patients with intractable epilepsy (with 71 contacts in amygdala, 31 in OFC and 43 in mPFC), we assessed the spectral dynamics and interactions between the amygdala, OFC and mPFC during an emotional picture viewing task. Task induced activity showed greater broadband gamma activity in the negative condition compared to positive condition in all the three regions. Similarly, beta activity was increased in the negative condition in the amygdala and OFC while decreased in mPFC. Furthermore, beta activity of amygdala showed significant negative association with valence ratings. Critically, model-based computational analyses revealed unidirectional connectivity from mPFC to the amygdala and bidirectional communication between OFC-amygdala and OFC-mPFC. Our findings provide direct neurophysiological evidence for a much-posited model of top-down influence of mPFC over amygdala and a bidirectional influence between OFC and the amygdala. Altogether, in a relatively large sample size with human intracranial neuronal recordings, we highlight valence-dependent spectral dynamics and dyadic coupling within the amygdala-mPFC-OFC network with implications for potential targeted neuromodulation in emotion processing.

## Introduction

The amygdala, orbitofrontal cortex (OFC) and medial prefrontal cortex (mPFC) are critical to emotional processing and regulation [1]. Human neuroimaging studies with functional magnetic resonance imaging (fMRI) provide substantial evidence for their involvement in emotion perception and evaluation [2, 3]. However, fMRI is dependent on the sluggish haemodynamic response (>3 s) and which remains an indirect measure of neuronal activity [4]. In contrast, intracranial electroencephalography (iEEG) records direct neuronal population level activity at high temporal resolution enabling insights into precise spectral dynamics. Thus, iEEG can potentially uncover specific frequency and timing differences underlying various emotions which may inform stimulation and targets for neuromodulation. Although a few studies have investigated emotional processing in the amygdala using iEEG, they have focussed predominantly on event related potentials (ERPs) [5, 6] with limited reporting of precise time-resolved spectral dynamics [7, 8]. Furthermore, only one iEEG study has examined OFC and mPFC activity in emotion processing [9] to the best of our knowledge and crucially none have investigated the interactions amongst this key emotion circuit with direct recordings. Consequently, direct evidence of valence-dependent coupling between the amygdala, the OFC, and mPFC remains poorly investigated in humans despite it being strongly implicated in affect and psychopathology [10].

The prefrontal cortex is believed to play a role in top-down modulation of amygdala activity providing contextualisation and evaluation of stimuli while the amygdala drives bottom-up salience processing [11]. However, the prefrontal cortex is a relatively large heterogenous structure with various distinct anatomical and functional divisions including the OFC and mPFC [12, 13]. Structurally, the amygdala is highly interconnected with both the OFC [14] and mPFC [15]. Tract tracing studies from non-human primate brains reveal afferent fibres from the OFC and mPFC terminating in the amygdala [15]. In turn, efferent fibres from the amygdala project to the OFC and mPFC [16]. Such structural connectivity constrains functional interactions between brain regions for fast and efficient transfer of information [17] highlighting the integrated coupling within this amygdala-OFC-mPFC network.

Functional evidence implicates a role for the OFC in emotional learning and evaluation [18] with its activation found in fMRI studies linearly related to subjective pleasantness [19]. Similarly OFC lesions impair subjective emotional states [20] and are associated with marked behavioural deficits [21]. The mPFC is believed to work in concert with the amygdala to tune the expression of emotions, such as fear and anxiety [22]. It has been hypothesised that the mPFC exerts inhibitory top-down control over amygdala activity, modulation of limbic and endocrine systems limiting their output and thus regulating emotion [23]. For instance, the mPFC is implicated in the regulation of fear expression after extinction [24]. In addition to regulating emotional responses, it is also implicated in integrating affective and contextual information [25]. Thus, an intricate network involving the OFC, mPFC and the amygdala is integral in emotion processing.

Due to the practical limitations of concomitant intracranial recordings from the amygdala, OFC and mPFC, the precise nature of responses and spectral dynamics to emotional stimuli and the interactions between these regions remain poorly characterised. Here, with iEEG recordings, we aimed to investigate the spectral dynamics and interactions of the amygdala with the OFC and mPFC during a static emotional picture paradigm. iEEG recordings provide a distinct advantage of accessing broadband gamma activity (BGA) (30–140 Hz) which is a spatially precise [26, 27], rich and a valuable measure associated with population spiking activity in the local field potential [28, 29]. More importantly, BGA is strongly correlated with blood-oxygen-level-dependent (BOLD) responses measured with fMRI [30, 31]. Thus, BGA provides a specific marker for comparing neuroimaging and electrophysiological data. Previous fMRI literature has demonstrated activation in the amygdala, OFC and mPFC with emotional picture tasks [32, 33]. Thus, we predicted task induced BGA in these regions. However, prefrontal cortices have been marred by the signal drop-out in fMRI studies particularly in the regions indicated here, at the air-tissue interface such as the orbitofrontal cortex [34, 35] making validation with direct neuronal recordings especially important. Furthermore, given prior reports of task induced theta activity in amygdala [8], we anticipated valence differences in this frequency range. Finally, we also hypothesised directed connectivity analyses to reveal top-down prefrontal influence over the amygdala.

## Materials and methods

### Participants

A cohort of 35 patients with medically intractable epilepsy, implanted with stereotactic electrodes for clinical evaluation at Ruijin Hospital, Shanghai, were included in the study. Inclusion criteria were 1) age: 18–60, 2) right-handed, 3) Montreal Cognitive Assessment (MoCA) score ≧24, 4) electrodes implanted at least one of the following areas: amygdala, OFC or mPFC. The choice of regions for electrode implantation was based on clinical criteria alone. Patient characteristics are provided in Table 1. Patients gave written informed consent to participate in the study and were free to withdraw from the study at any time. The study was approved by the Human Research Ethics Committees of the Ruijin Hospital, Shanghai, China and performed in agreement with the Declaration of Helsinki.

**Table 1:** Patient demographics.

	PID	Sex	Age (yrs)	Handedness	Diagnosed epileptogenic zone
1	P1	F	53	R	right hippocampus, right amygdala
2	P2	F	20	R	right hippocampus, right amygdala
3	P3	F	23	R	right hippocampus, right amygdala
4	P4	M	32	R	left hippocampus, left amygdala
5	P5	F	24	R	Right insula
6	P6	M	32	R	right hippocampus, right amygdala
7	P7	F	29	R	left hippocampus
8	P8	F	20	R	right hippocampus, right amygdala, right anterior temporal lobe
9	P9	M	19	R	right hippocampus, right amygdala
10	P10	M	18	R	right occipital lesion
11	P11	F	24	R	left anterior temporal lobe
12	P12	M	19	R	left hippocampus, left amygdala
13	P13	M	24	R	left hippocampus, right frontal
14	P14	F	23	R	left hippocampus
15	P15	M	38	R	right hippocampus
16	P16	M	24	R	Left frontal
17	P17	M	21	R	right inferior frontal gyrus
18	P18	F	36	R	left cingulate
19	P19	F	30	R	Left cingulate
20	P20	M	39	R	left hippocampus, left amygdala
21	P21	M	20	R	occipital lobe
22	P22	F	30	R	right hippocampus, right amygdala
23	P23	F	30	R	right hippocampus, right amygdala
24	P24	M	43	R	right anterior temporal lobe, right hippocampus, right amygdala
25	P25	M	29	R	right anterior temporal lobe
26	P26	F	46	R	right anterior temporal lobe
27	P27	M	44	R	left anterior temporal lobe
28	P28	F	25	R	right occipital lobe
29	P29	M	30	R	right frontal lobe
30	P30	F	36	R	left hippocampus, left cingulate
31	P31	M	31	R	bilateral anterior limb of internal capsule
32	P32	M	21	R	left temporal lobe
33	P33	M	23	R	left anterior temporal lobe and left orbitofrontal cortex
34	P34	M	19	R	left temporal lobe
35	P35	M	26	R	right frontal lobe and right hippocampus

### Data acquisition

#### Stereo-EEG recordings

Implantation of intracerebral electrodes with multiple contacts (Huake-Heushang SDE 08, 8–18 contacts, electrode diameter: 1 mm, intercontact spacing 1.5 mm) was performed with a stereotactic procedure and planned individually based on the likely seizure onset zone inferred by the clinicians (SZ, WL and CC) from non-invasive pre-operative studies. iEEG signals sampled at 1 kHz were recorded on a Brain Products amplifier with 32 channel (for first 3 patients) and 64 channel capacity for others. All experimental task data were processed off-line using EEGLAB [36] and custom routines programmed in MATLAB (MathWorks, MA, USA). Recordings were down-sampled to 500 Hz and band-passed filtered between 0.1 and 195 Hz using a zero-phase lag filter (FIR). Power line 50 Hz noise and its harmonics were notch filtered. Data were referenced with bipolar re-referencing scheme.

**Fig. 1:**
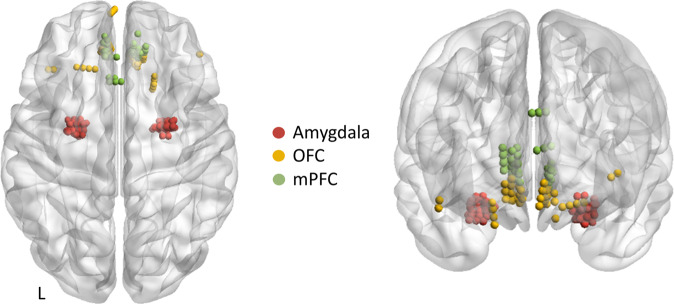
Electrode channel localisation rendered onto MNI space. Colours indicate contacts in each region. MNI coordinates of each contact are given in supplementary table 1. OFC orbitofrontal cortex, mPFC medial prefrontal cortex, L left.

#### Electrode localisation (Fig. 1)

Intracranial electrodes were localised on a 3-d MNI brain template using a fusion of pre-operative magnetic resonance (MR) and post-operative computed tomography (CT) scans using Brainstorm [37]. We started by co-registering the CT image to MR T1 image and normalising them to the MNI template. The co-registered and normalised CT and MR images were then visually inspected for the anatomical location of the electrode channels. The electrodes channels were also assessed on the AAL atlas [38] to confirm their location in the anatomical regions of interest. The electrode channels are shown in MNI space (Fig. 1). The implantation maps of the electrode contacts localised by the neurosurgeon were also used to corroborate the presence of contacts in the anatomical regions of interest. Only electrode contacts from the non-epileptogenic zone as diagnosed by the clinicians were used for analyses. Overall, there were 27 patients with a total of 71 contacts in the amygdala, 11 patients with 31 OFC contacts and 13 patients with 43 mPFC contacts. Three patients contributed to amygdala contacts on both the hemispheres. All the MNI coordinates of the contacts from each patient are provided in Supplementary Table 1. For connectivity analyses 74 unique contact pairs were used for the amygdala-OFC dyad, 84 unique pairs for the amygdala-mPFC dyad, and 83 unique pairs were used for the OFC-mPFC dyad.

#### Experimental paradigm

Emotional pictures from the International Affective Picture System (IAPS) [39] were used as stimuli. The paradigm has been described in detail in a previous publication [40]. Briefly, a total of 90 different images were selected, with 15 images (5 per category) rated for valence and 15 images (5 per category) for arousal using a sliding scale (0–100). The picture stimuli were presented for 2 s each and the intertrial interval jittered from 1 to 1.5 s. Participants were presented the stimuli on a laptop with screen size of 14 inches placed ~60 cm in front of them. The experimental paradigm was presented using Psychtoolbox [41]. 5 images were randomly selected from each valence condition to be rated by the patient on a valence (0 (unpleasant)–100 (pleasant)) and arousal scale (0 (calm)–100 (excited)).

### Data analysis

Data trials corresponding to 500 ms baseline (consisting of a fixation cross preceding the stimulus during the inter-trial interval) and 2000 ms post-stimulus onset were used for analyses. Visual inspection was also employed to identify inter-ictal spikes, defined as paroxysmal discharges lasting less than 250 ms [42] and such trials were excluded from further analysis.

#### Event related spectral perturbation (ERSPs)

ERSP measures variations in power spectrum at specific frequency ranges of ongoing rhythms at specific periods of time and frequency range normalised by the baseline [43]. We used a baseline of 500 ms prior to the onset of the affective image and post-stimulus onset window of 2000 ms and employed fast-fourier transform (fft) to compute ERSPs in the frequency range of 2–140 Hz in EEGLAB. The outputted ERSPs had temporal resolution of 2 ms and frequency resolution of .2 Hz. These maps were first computed for each electrode contact (frequencies x time) and data from each contact (belonging to a region of interest) compiled producing 3D data (frequencies × time × contacts). To avoid potential multiple comparison issues with three valence conditions, we first subtracted averaged ERSP maps of each electrode contact for neutral conditions from positive and negative conditions. Thus, we use ERSP maps for positive (relative to neutral) and negative (relative to neutral) condition for statistical analysis via permutation testing described below.

#### Statistical testing

To compare time frequency maps (ERSPs) between positive (relative to neutral) and negative conditions (relative to neutral), a nonparametric cluster-based permutation approach [44]. This approach specifically corrects for multiple comparison issues in multidimensional data such as time-frequency decompositions. It enables identification of clusters (time window and frequency bins) with significant differences in the power changes induced by the presentation of pictures of different emotional valence. For permutations, the original paired samples for each region (condition ERSPs for each contact) were randomly permuted 1000 times such that each pair was maintained but its assignment to the condition (positive or negative) changed to create a null-hypothesis distribution. For each permutation, a *t* value is computed for each sample data point and clusters with *p* < 0.01 are identified. Such samples are clustered in connected sets and cluster level statistics calculated [44]. We restricted the size of cluster >500 to find robust condition differences and to avoid small potentially meaningless clusters. Average power in the significant clusters were further compared using post-hoc paired t-tests with multiple comparison false discovery rate (FDR) correction [45]. The condition differences were plotted raw time frequency plots (i.e., without subtraction of neutral condition) to appreciate early task induced activity and condition differences. For association of the mean of significant clusters in traditional frequency ranges from each patient with corresponding valence ratings, Pearson’s correlation coefficient was computed to investigate significant correlation. Note that for these associations, valence ratings for positive and negative conditions were computed relative to neutral valence ratings. For patients with multiple contacts in the region of interest, we averaged the significant cluster for those contacts. Valence ratings for one patient with amygdala contacts and three who had concomitant OFC and mPFC contacts could not be acquired.

#### Time varying coherence

Coherence is a measure of undirected functional connectivity which provides a frequency domain measure of the interdependence between signals [46]. Its magnitude varies between 0 and 1 with a value of 0 again indicating a complete absence of synchronisation and 1 indicating perfect synchronisation. We used linear coherence as employed in EEGLAB. We used all possible combinations of the contact-pairs in a dyad for computing coherence and subsequent connectivity analyses. Same sided hemisphere contacts were used where available. Concomitant contacts in all three regions were only available for six patients. The small number of patients would constrain the generalisability and interpretation of results. Consequently, we utilised data from patients with all possible dyadic pairs to improve generalisability. Overall, there were nine patients for amygdala-OFC dyad with a total 77 unique contact pairs, 10 patients for amygdala-mPFC dyad with 84 contact pairs and 12 patients for OFC-mPFC dyad with 83 contact pairs.

#### Spectral Granger causality (spectral GC)

Time domain Granger Causality (GC) assesses conditional (asymmetric) dependencies between two signals assessing if information from one signal is useful to predict the other [47]. GC has also been extended to the frequency domain (spectral GC), to characterise the frequency content of directional dependencies between the signals. Specifically, spectral GC uses Fourier methods to examine “granger-causality” in the spectral domain [48, 49] and measures the fraction of the total power at a particular frequency contributed by each signal. We employed the multivariate granger causality toolbox [50] to compute the spectral GC. The model order *m* was determined according to the Akaike information criterion, which is a trade-off between spectral resolution and complexity. Model orders were estimated for each patient and varied from 7 to 12. Spectral GC estimates were considered significant if they exceeded the 99.9% confidence interval established by permutation testing (1000 randomisations). The permutation statistics were computed by randomly shuffling the time series in blocks, which take the computed autocovariance lags into account [50]. Spectral granger causality was computed for each unique pair of contacts. Thus, each pair have two spectral GC curves associated with them (in each direction for e.g., in amygdala-OFC dyad: amygdala to OFC and OFC to amygdala). The spectral GC curves for all the pairs in a dyad were averaged and plotted with standard error of mean shown as the shading.

#### Dynamic Causal Modelling (DCM)

DCM provides a computational framework to infer effective connectivity which quantifies the influence one neural system exerting over another [51]. DCM is a Bayesian framework to infer hidden neuronal states from the observed neurophysiological data features [52] in contrast to GC which uses observed signals. For this it uses a biophysical neuronal model, and the observation model, which describes neuronal source activity translates to data acquired at the sensor level [53]. Furthermore, DCM employs hypothesis driven model specification of connectivity between the brain regions allowing the effect of experimental conditions to be modelled as modulators of the connectivity between the regions [51]. We employed DCM for cross spectral densities (CSD) which uses cross-spectrum as the data feature to be modelled as implemented in SPM12 [54] focussing on 2–45 Hz. We further used the local field potential (lfp) neural mass model (biophysical neuronal model) which is the modification of the Jansen and Rit neuronal model [55] incorporating local (within-region) as well as between region connectivity drives. Valence modulation was entered as binary values; +1 for positive, 0 for neutral and −1 for negative pictures. The model space for DCM encompassed all possible (forward and backward) connections between the amygdala - OFC, amygdala - mPFC, and OFC - mPFC and all possible manners in which the valence of the stimuli modulated these connections.

For group model comparison, Bayesian model selection (BMS) with random effects was employed to select the winning model at the group level. This approach uses a variational bayes method that balances the posterior likelihood of a model with its complexity in order to identify the model with the highest exceedance probability (the “winning model”) [51].

## Results

The data were subjected to a series of task induced time frequency decompositions, functional and effective connectivity analytical approaches to quantify the local responses in each region, condition differences as well as statistical and dynamic interdependences between the regions.

### Task induced activity

**Fig. 2:**
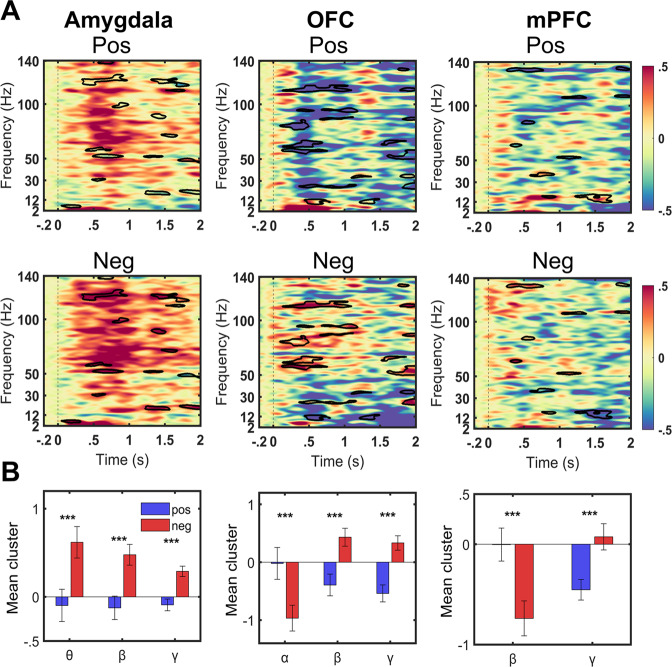
Task induced activity with condition differences. **A** Grand averaged event related spectral perturbation (ERSP) maps for the amygdala (left), the orbito-frontal cortex (OFC) (middle) and medial prefrontal cortex (mPFC) (right) for positive (top) (Pos: relative to neutral) and negative (bottom) (Neg: relative to neutral) conditions. Warmer colours denote task-induced power increases from the baseline, while cooler colours refer to power decreases from the baseline. Significant differences between conditions shown in black outlines. Statistics were computed on time-frequency data as relative change to neutral condition (see methods). **B** Bar plots corresponding each column in **A** showing the group mean of the significant frequency clusters on post-hoc t-tests after multiple comparison corrections. Errors bars indicate standard deviation. ***p_FDR_ < .001.

The amygdala showed greater early theta (0–1 s) and late beta (1–2 s) and BGA activity to negative compared to positive stimuli (Fig. 2A*left, B left*) (Theta (positive: −0.10 (0.18), negative: 0.62 (0.17), *t*_*70*_ = *−3.56, P*_*FDR*_ = 0.0006), Beta (positive: −0.12 (0.13); negative: 0.48 (0.12); *t*_*70*_ = *−4.72, P*_*FDR*_ = 0.00001), BGA (positive: −0.13 (0.05), negative: 0.48 (0.06), *t*_*70*_ = *−4.71, P*_*FDR*_ = 0.0000008)).

Similarly, the OFC also showed higher beta activity and further showed lower alpha activity in the contrast of negative compared to positive stimuli (Fig. 2A*middle, B middle*) (Alpha (positive: −0.02 (0.28); negative: −1.01 (0.22); *t*_*30*_ = 4.56, P_*FDR*_ = 0.00008), Beta (positive: −0.39 (0.19); negative: 0.43 (0.16); *t*_*30*_ = *−4.31, P*_*FDR*_ = 0.0001), BGA (positive: −0.54 (0.15), negative: 0.33 (0.12), *t*_*30*_ = *−5.50, P*_*FDR*_ = 0.000005)). In contrast, the mPFC showed the opposite direction of activity with lower beta for negative relative to positive stimuli (Fig. 2A*right, B right*) (Beta (positive: −0 .003 (0.16); negative: −0.74 (0.17); *t*_*42*_ = 4.72, P_*FDR*_ = 0.00002, BGA (positive: −0.45 (0.10), negative: 0.07 (0.13), *t*_*42*_ = −4.76, P_*FDR*_ = 0.00002)*)*. Significant clusters overlaid on time frequency contrasts of positive > neutral and negative > neutral are shown in Supplementary Fig. 1.

### Beta activity of amygdala is associated with valence ratings

**Fig. 3:**
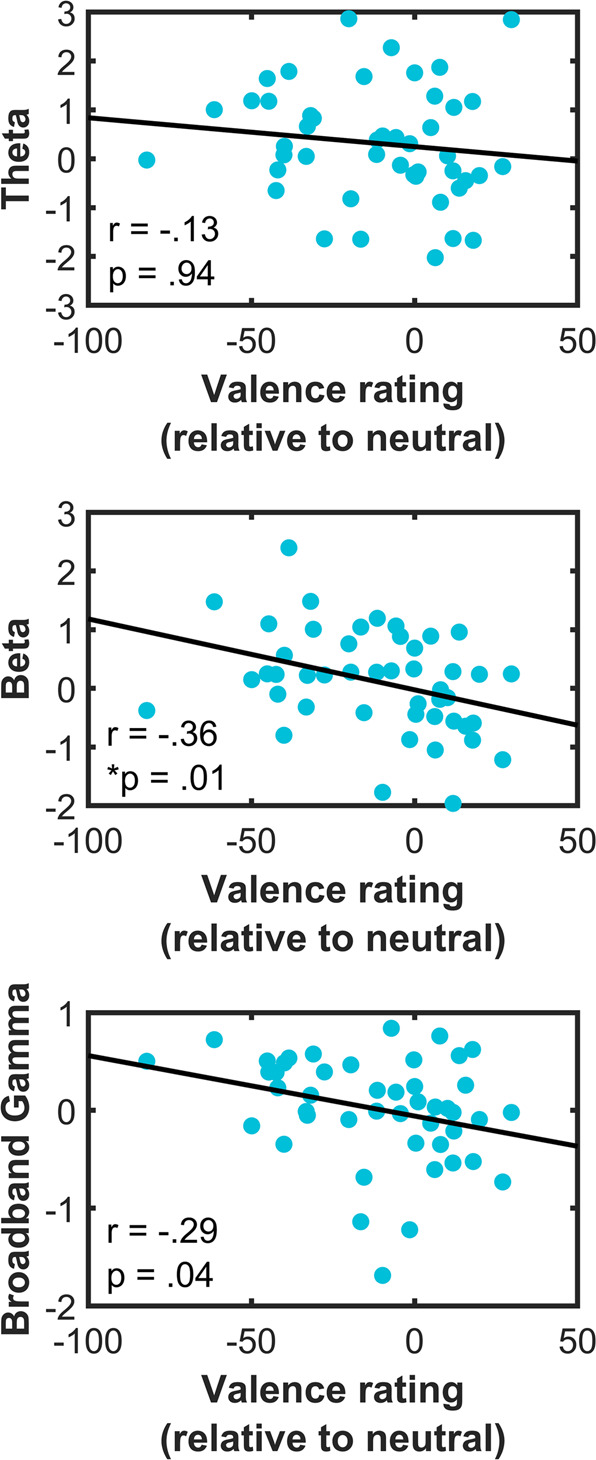
Association of amygdala activity with valence ratings. Mean of significant theta cluster (top) showed no significant association with valence ratings whilst beta activity (middle) showed significant negative relationship with valence ratings i.e greater beta activity was associated with greater negative valence. Broadband gamma (bottom) also showed negative relationship with valence ratings, but which did not withstand multiple comparisons testing. Multiple comparison correction at p_FDR_ < 0.05. Valence ratings computed relative to neutral valence ratings (see methods). Unadjusted *p* values are shown.

We then assessed the relationship between mean of significant clusters in each frequency band and valence ratings on an exploratory basis (Fig. 3). Beta activity of amygdala was significantly negatively correlated with valence ratings i.e., greater beta activity for negative valence and decreased beta activity for positive valence. Given prior evidence for laterality of amygdala function in emotional processing [56, 57], we further computed correlations of left and right amygdala activity separately with valence ratings (Supplementary Fig. 2). No significant associations were found. There was also no significant relationship between the frequency band significant clusters in OFC and mPFC with valence ratings (Supplementary Fig. 3).

### Connectivity analysis

Although there are numerous connectivity metrics for EEG/iEEG data, we chose a hierarchical approach of using undirected (coherence), directed (spectral GC) and effective connectivity (DCM).

#### Coherence

**Fig. 4:**
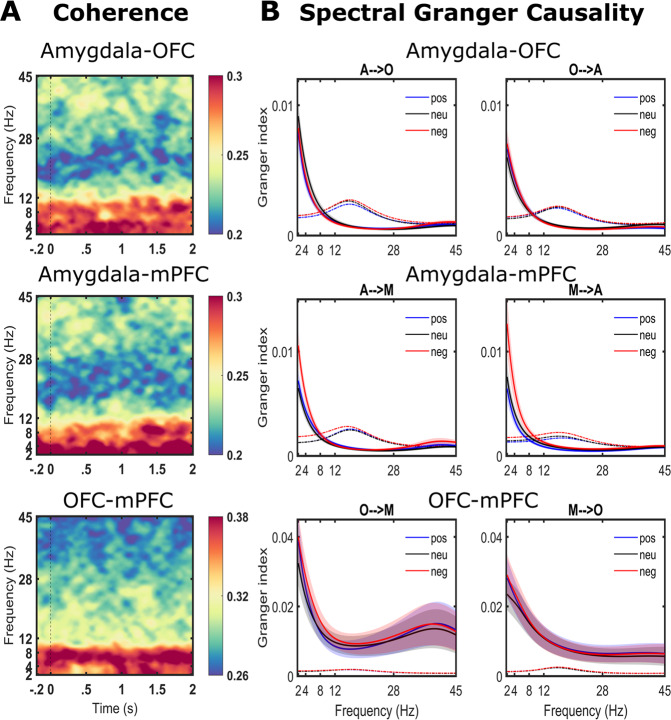
Functional connectivity. **A** Grand averaged time varying coherence plots (averaged over all conditions) highlights strong connectivity in the lower frequency range of <12 Hz. **B** Spectral granger causality (sGC) results for the three valence conditions (Positive: pos, Neutral: neu, Negative: neg). Top: Amygdala (A)-orbitofrontal cortex (O), middle: Amygdala (A)-medial prefrontal cortex (M), bottom: OFC (O)-mPFC (M). Dotted lines show 99.9 % confidence interval (CI) after 1000 permutation testing. sGC analyses confirm the results from coherence analysis suggesting predominant connectivity in the low frequency range.

Coherence is an undirected measure of functional connectivity which we first used to characterise the predominant frequency range of communication between the dyadic pairs of the amygdala, the OFC and mPFC. Our main objective was to quantify the frequency range of communication and we did not have hypotheses for statistical differences in time varying coherence. Hence, we concatenated all the condition types and estimated coherence. All three regions showed predominantly higher coherence in the lower frequency range (<12 Hz) (Fig. 4A). We further explored coherence differences between the dyadic pairs with identical statistical testing method as employed for ERSP maps (see methods). No differences in coherence were observed between amygdala-OFC Vs amygdala-mPFC. However, two main frequency ranges showed strong coherence differences when testing OFC-mPFC Vs amygdala-mPFC and OFC-mPFC Vs amygdala-OFC: beta and theta\alpha (Supplementary Fig. 4). The coherence in both these frequencies was higher for OFC-mPFC dyad when compared to amygdala-mPFC and amygdala-OFC dyad. Given the proximity of OFC and mPFC, both being part of prefrontal cortices, and their role in higher order cognition, it thus seems likely that the synchronisation in their activity is higher spanning broader frequencies.

#### Spectral Granger Causality (GC)

Spectral GC confirmed the low frequency range of communication as seen from coherence results, but which was bidirectional for amygdala-OFC and amygdala-mPFC while broad frequency range bidirectional communication was found for OFC–mPFC pair (Fig. 4B). Granger indices in the lower frequency range of <12 Hz withstood the threshold on 99.9% confidence interval obtained by 1000 permutations. For the OFC-mPFC interaction, granger indices in 2–45 Hz were significant. Spectral GC averaged over conditions are shown in Supplementary Fig. 5.

#### Effective connectivity

**Fig. 5:**
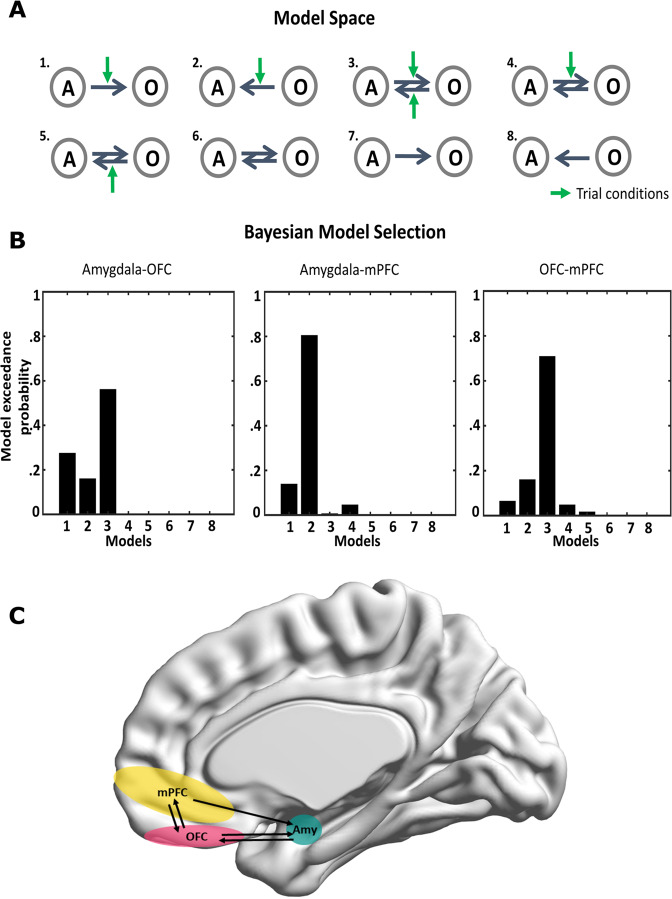
Dynamic causal modelling (DCM) (2–45 Hz). **A** The model space comprised eight models, with all possible connections and task modulation effects influencing the connectivity between the dyads on which DCM was employed. Only models for amygdala (A) and orbitofrontal cortex (O) are shown here as an example. Each corresponding forward connection has a corresponding backward connection. Only forward connections are shown for simplicity. The green arrow indicates task modulation (positive, neutral and negative valence pictures). For the amygdala-medial prefrontal cortex (mPFC) and the orbitofrontal cortex (OFC)-mPFC dyads, model space was identical as with the amygdala-OFC dyad with eight models **B** Bayesian model selection for the amygdala-OFC (left) and the OFC-mPFC (right) connectivity identified model 3 (bidirectional connectivity) as possessing the highest exceedance probability of 58% and 72% respectively whereas for the amygdala-mPFC dyad (middle), model 2 (top-down mPFC to amygdala connectivity) was the winning model with probability of 81%. **C** Illustration of effective connectivity between amygdala (Amy)-OFC-mPFC. Note that this illustration is based on the results from dyadic interactions and not on three node network.

The model space with various possible ways of valence condition modulating the connectivity is shown in Fig. 5A. For the amygdala-mPFC dyad, the best fitting model showed that emotional valence modulated the unidirectional connection from mPFC to amygdala with more than 80% exceedance probability (Fig. 5B middle). In contrast, the model with the highest exceedance probability for the amygdala-OFC dyad and OFC-mPFC dyad showed that emotional valence modulated bidirectional connections (Fig. 5B*left and right*, respectively).

## Discussion

Using human intracranial EEG recordings from deep brain structures, we demonstrate that viewing of emotional pictures evokes a complex activity over frequencies in the amygdala, the OFC and mPFC. BGA was greater in the negative condition in all the three regions. Spectral dynamics in lower frequencies (2–30 Hz) showed condition differences in beta frequency range as a common differentiating frequency. Undirected and directed connectivity analyses between these dyads indicated interactions predominantly in the low frequency range (<12 Hz). Ultimately computational analyses of effective connectivity revealed unidirectional top-down influence from mPFC to the amygdala, with a bidirectional coupling observed between the amygdala-OFC and the OFC-mPFC dyad.

### High frequency activity

BGA is a precise measure of local neuronal activity [27] that has also been associated with the BOLD signal from fMRI studies [29, 31]. BOLD activation maps have often implicated the amygdala, OFC and mPFC in affective studies especially with picture stimuli [32, 58]. These prior findings had led us to expect BGA response in all the three regions. Amygdala showed increased BGA in both positive and negative conditions. This is consistent with other iEEG studies which also found BGA in the amygdala for emotional stimuli [8]. The OFC and mPFC showed greater BGA in negative condition when compared to positive condition. Furthermore, BGA was decreased in positive condition relative to baseline. Since BOLD activation have been reported with positive emotions in these regions, reduced BGA in positive condition for OFC and mPFC raise important questions regarding the association of BOLD activity with local field potentials. LFP-BOLD coupling is complex and perhaps different frequencies may uniquely contribute to the coupling. For instance, BGA is positively correlated with the BOLD signal [59], but alpha frequency has also been shown to be negatively associated with it [60]. Furthermore, various analytical choices with iEEG such as the referencing scheme, intercontact spacing, spatial spread of lfps and/or various subtle dependencies on different methods of fMRI pre-processing may subtly affect lfp-BOLD coupling. Further investigations to characterise the association of lfps and BOLD, especially in higher order regions, are thus warranted.

### Low frequency spectral dynamics

In the low frequency domain, task induced responses in the amygdala, the OFC and mPFC showed unique frequency clusters which differentiated positive and negative valence conditions. Amygdalar iEEG studies characterising ERP responses [6, 61] show greater responses to negative conditions. However, precise time-frequency dynamics and valence differences with IAPS stimuli have not been reported to our knowledge. Here, we thus first shed light on the precise spectral dynamic responses of the amygdala in emotional picture viewing. Critically, dissociable responses as a function of valence were observed in the amygdala with negative valence conditions showing increased activity in early theta (0–1 s) and late beta (1–2 s) when compared to positive stimuli. Theta-alpha power in the amygdala for emotional stimuli has been reported previously with short (1.5 s) dynamic facial emotional videos [8] which were demonstrated with power profiles without time frequency decompositions and thus timing differences were not investigated. Furthermore, theta range communication of amygdala with hippocampus in emotional processing [8] and that with mPFC in a condition-extinction paradigm has been shown in iEEG studies [9]. Our results thus highlight a consistent role of theta activity in emotional evaluation at amygdala.

We further show amygdalar beta activity was associated with subjective valence ratings. Specifically, greater beta power was associated with greater negative valence ratings. Past studies have demonstrated amygdala activity differentiating valence [62, 63]. Interestingly, Scangos et al. demonstrated beta power of the cortico-limbic regions including amygdala as predictive of depression severity [64]. Furthermore, greater beta frequency coherence connectivity between amygdala and hippocampus has also been associated with worsening mood [65]. Thus, beta frequency of the amygdala may be an important marker in emotional evaluation which future studies with neuromodulation can leverage.

Lower alpha activity and greater beta in the OFC for negative condition and lower beta activity in the mPFC for negative stimuli differentiated negative from positive images. Previous findings suggest early OFC and mPFC responses in emotional perception [66]. Limited studies exist characterising iEEG responses from the OFC. Late (1–2 s) ERD has been demonstrated in various deep brain stimulation (DBS) studies from subgenual anterior cingulate cortex in the beta frequency range [67] and in alpha range at from subthalamic nuclei [40] and habenula [68]. Prior reports from iEEG studies have also demonstrated OFC response latency to aversive cues similar to that of amygdala (~200 ms) [69]. Given that the OFC and mPFC have widespread connection with the basal ganglia and anterior cingulate, our results thus indicate beta activity as potentially specific for emotional differentiation.

### Functional and effective connectivity

Much literature has focussed on the amygdala responses to emotional perception; however, there is increasing interest of its interaction with key emotional circuitry such as with temporal pole [70], OFC [71], and mPFC [9]. Our results of bidirectional connectivity between the amygdala and the OFC during affective stimuli are consistent with causality analyses undertaken with fMRI showing significant bidirectional connectivity between them [72]. Given the role of the OFC in reward learning and stimulus value, reciprocal interactions between amygdala and OFC may be integral to the integration of emotional relevance to complex emotional perception to generate emotional value. Previous studies identify the OFC as a key node within an emotional network activated by anticipation of aversive events [10]. Consequently the OFC is thought to integrate multimodal sensory information and guide emotion-related decisions by evaluating expected outcomes [18]. Neuromodulation in patients with epilepsy by stimulating the OFC has also been shown to improve mood [73] and modulate subjective valence [74] thus furthering these claims of the OFC as integral to affective cognition.

Unlike the bidirectional influence between the OFC and the amygdala, mPFC is believed to function in regulation of affective processing specifically in a unidirectional top-down manner to inhibit negative emotional processing in the amygdala and prevent excessive physiological and behavioural responses [75]. Converging studies support a role for mPFC in reducing conditioned fear responses via its projections to the amygdala [76]. Evidence from rodent experiments provide direct physiological support that mPFC reduces fear responses by reducing amygdala output [77]. Consequently mPFC activity has been strongly linked with reappraisal of emotions especially of fear responses [78]. Emotional behavioural deficits such as aggressiveness is also related to reduced amygdala–mPFC functional coupling in fMRI studies [79]. mPFC may thus perform a generic negative emotion inhibitory function that can be recruited by other regions. Our results are consistent with a theoretical model of mPFC whereby it regulates emotional states and generate affective responses and autonomic responses in an optimised fashion via its influence on the amygdala. Interestingly, pathological hyperconnectivity between mPFC and the amygdala has been shown in patients with depression [80]. More significantly, weakening this connectivity with trans-cranial direct current stimulation in patients with depression enabled them to regulate emotion efficiently [81].

Importantly, the directional influence of mPFC over amygdala was only apparent from effective connectivity analysis of DCM whilst spectral GC revealed bidirectional influence between them. GC and DCM both measure directed causal interactions fundamentally differing in the fact that GC models dependency among observed signals (EEG/MEG/lfp) while DCM models coupling among the hidden states generating these signals [82]. GC and DCM are thus complementary approaches, but which may not always preclude divergent results. Some consistency in convergent results have been shown with GC and DCM (for instance [70]). In fact, even in our results here, the bidirectional coupling between amygdala-OFC and OFC-mPFC is consistent with both GC and DCM analyses. This probably points to the fact that temporal precedence in the two dyad pairs of amygdala-OFC and OFC-mPFC was the primary driver of coupling with no bearing of how the valence conditions modulated the connectivity. In contrast, for the amygdala-mPFC dyad, the valence condition modulation of the connectivity had a dominant bearing in addition to the temporal precedence in the data reflected in the winning model. Another advantageous feature of DCM is its ability to provide mechanistic insights by employing neurobiologically inspired model of neuronal cellular architecture (lfp model [83]) to study the causal influence between the region’s activity. Specifically, the use of a biophysically inspired neuronal connectivity model is based on bottom-up and top-down influences in the brain [84]. Such biophysical neuronal models incorporate the functional architectural principles of brain with bottom-up and top-down connectivity [85] thus providing an accurate framework for studying causal connectivity.

### Limitations and conclusion

There are several caveats of the study. First, our data comes from patients with epilepsy. However, we employed strict quality control such as utilising the data from non-epileptogenic zones, visual inspection, and trial rejection to mitigate possible effects of epileptiform activity on our task findings. Second, the OFC and mPFC are functionally and anatomically large regions. Specifically, OFC is divided into medial and lateral portions and mPFC in dorsolateral and ventro-medial regions [86]. Furthermore, the amygdala is also composed of various subnuclei [1]. Consequently, subtle differences may underlie the task induced responses and their connectivity based on the precise location of the contacts. Given the unique implantation schemes in each patient, a large sample size study with specific distinct sub-region profile is challenging. A larger sample size with concomitant contacts in all the three regions is needed to robustly estimate the effective connectivity in a three-node network. In the current study, we utilised data from patients with all possible dyadic pairs to improve generalisability of the results. However, for completeness we did undertake three node DCM analyses with limited subjects and limited model space, which showed a bidirectional connectivity model as the winning model (Supplementary Fig. 6), which showed unidirectional influence from mPFC to amygdala and bidirectional connectivity between OFC-mPFC. One differing characteristic of the winning 3 node model was the amygdala to OFC influence. It is possible that different and limited subjects may have yielded differing results which warrant further investigations. Finally, with our DCM analyses, the model exceedance probability of the winning model for the amygdala-OFC dyad might be considered on lower probability than required to unequivocally establish the influence between the OFC and the amygdala. One reason for this might be the heterogenous location of the OFC contacts. However, the winning model had an exceedance probability clearly greater than the other models inclusive of null models thus suggesting its superiority in explaining our data.

In sum, this work highlights detailed spectral responses of the amygdala, OFC and mPFC differentiating valence conditions. We demonstrate for the first time using direct intracranial recordings evidence for a top-down influence of mPFC over the amygdala and bidirectional connectivity between the OFC and the amygdala coupling during emotional processing. This converges with research showing that spatially diverse and complex neural dynamics support emotional and cognitive functions in this circuitry [87]. A core neural dynamic involving the amygdala, the OFC and mPFC thus underlies the emotional evaluation. Future work could build on this and explore dynamic modulations and the effects of stimulation between these nodes and circuit with implications for neuromodulation targets for affective pathologies.

## Supplementary information

Supplementary

## Data Availability

The data for this project were acquired from patients undergoing clinical care and consenting for additional research protocols. Researchers wishing to access these data will require local ethics approval and a data sharing agreement with Ruijin Hospital, Shanghai, China
