# Supplementary material for: Frequency dependent emotion differentiation and directional coupling in amygdala, orbitofrontal and medial prefrontal cortex network with intracranial recordings
Source: Mol Psychiatry. 2022 Dec 2;28(4):1636–46. doi: 10.1038/s41380-022-01883-2 (PMC10208964; doi:10.1038/s41380-022-01883-2)
Supplement: Supplementary file 1 — Supplementary [file 41380_2022_1883_MOESM1_ESM.pdf]

Supplementary Information

Frequency dependent emotion differentiation and directional coupling in amygdala, orbitofrontal and medial prefrontal cortex network with intracranial recordings

Saurabh Sonkusare, Ding Qiong, Yijie Zhao, Wei Liu, Rocky Yang, Alekhya Mandali, Luis Manssuer, Chencheng Zhang, Chunyan Cao, Bomin Sun, Shikun Zhan, and Valerie Voon

STable 1: MNI coordinates of contacts

**Left Hemisphere – MNI coordinates of contacts**			
PID	Amygdala	Orbitofrontal cortex (OFC)	Medial prefrontal cortex (mPFC)
P1	-19.1, -1.0, -18.1<br>-22.9, -0.8, -18.4<br>-26.5, -0.4, -17.8		
P2	-23.3 -8.8 -16.1		
P4			-7.1 41.5 -3.7<br>-7.1 44.2 -0.8<br>-7.1 46.8 2.0
P6	-26.2 -4.6 -21.4<br>-29.4 -4.8 -20.5		
P7		-6.5 36.7 -15.3<br>-6.9 37.4 -11.3	-7.4 38.1 -7.3<br>-7.8 38.7 -3.3<br>-8.3 39.4 0.6<br>-8.7 40.1 4.6
P11	-23.4 -5.2 -21.2<br>-26.9 -5.2 -20.6		
P12		-2.7 64.4 -16.4<br>-3.5 63.6 -12.7<br>-4.3 62.8 -8.9<br>-5.1 61.9 -5.2	0.9 22.8 29.1<br>-2.4 22.7 29.5<br>-5.8 22.6 29.9
P14	-21.3 -1.6 -16.9<br>-25.0 -1.6 -16.4<br>-28.6 -1.7 -15.9		
P15	-21.9 -6.5 -19.1<br>-25.3 -6.5 -18.3		
P16	-21.4 -8.0 -11.6<br>-24.9 -7.8 -11.3		
P17	-22.5 1.2 -22.8<br>-26.1 1.0 -21.8<br>-29.7 0.8 -20.8		
P21	-21.5 -4.9 -18.7<br>-25.0 -5.0 -17.9		

	-28.4 -5.1 -17.1		
P22		-5.5 36.9 -14.5<br>-6.1 39.3 -11.4<br>-6.7 41.7 -8.2	-7.3 44.1 -5.0<br>-7.9 46.4 -1.9<br>-8.5 48.8 1.2
P23	-21.7 -4.7 -20.3<br>-25.4 -4.6 -19.5<br>-29.0 -4.5 -18.7		
P24	-23.2 -6.8 -18.0<br>-26.6 -6.9 -17.6	13.7 35.3 -13.0<br>13.9 36.4 -9.3<br>14.1 37.4 -5.6	-8.5 44.6 -3.4<br>-9.3 45.2 0.2
P26	-21.6 -5.7 -19.7<br>-25.2 -5.2 -19.1<br>-28.8 -4.8 -18.6		
P30	-25.0 -1.0 -25.1<br>-28.8 -1.3 -24.2		
P31	-20.5 -2.6 -22.8<br>-24.0 -2.5 -22.0<br>-27.4 -2.5 -21.3		
P32	-20.6 -5.3 -17.4<br>-23.8 -5.4 -16.8<br>-27.1 -5.5 -16.2<br>-30.4 -5.6 -15.7	-4.8 41.4 -15.1<br>-5.5 42.9 -11.9<br>-6.2 44.4 -8.8	-2.7 24.8 29.2<br>-6.0 25.1 29.6
P34	-24.3 -4.3 -23.7<br>-27.8 -4.4 -23.2		
**Right Hemisphere– MNI coordinates of contacts**			
PID	Amygdala	Orbitofrontal cortex (OFC)	Medial prefrontal cortex (mPFC)
P4	24.2 -2.9 -17.3<br>27.6 -2.8 -17.2<br>31.0 -2.7 -17.1	6.7 35.4 -14.9<br>6.9 36.7 -11.0	7.5 40.7 0.5<br>7.7 42.1 4.4<br>7.9 43.4 8.3<br>8.1 44.7 12.2
P5	24.2 -2.9 -17.<br>27.6 -2.8 -17.2<br>31.0 -2.7 -17.1		
P8	-29.7 -6.2 -12.9		
P9	26.7 -3.4 -19.3<br>30.0 -3.1 -18.9		
P10	19.1 -4.6 -16.9<br>22.7 -4.5 -16.7<br>26.2 -4.3 -16.6<br>29.8 -4.1 -16.4	5.5 37.8 -12.9<br>6.1 38.5 -8.8	10.6 36.4 -3.7<br>10.7 38.1 -0.4<br>10.9 39.9 2.9<br>11.0 41.7 6.2
P11	28.5 -3.7 -21.1<br>32.0 -3.7 -20.7		
P12	24.2 -2.0 -15.1<br>27.6 -2.5 -15.1<br>30.9 -3.0 -15.0		
P13	20.5 -3.1 -15.9<br>24.0 -3.2 -15.6<br>27.6 -3.3 -15.4<br>31.1 -3.4 -15.1		

P15			8.6 38.1 -2.8<br>9.3 39.5 0.8<br>10.1 40.8 4.4
P17	23.0 -3.1 -14.2<br>26.7 -3.2 -14.0<br>30.5 -3.2 -13.8		
P18	21.0 -3.7 -16.1<br>24.9 -3.7 -16.0<br>28.8 -3.7 -15.9		
P20			-1.6 36.9 10.9<br>-5.1 37.1 11.9<br>-8.5 37.2 12.9
P21	23.2 -0.3 -19.3<br>26.7 -0.3 -19.0<br>30.2 -0.3 -18.7		
P22	22.9 -0.0 -21.4<br>26.8 0.0 -21.2<br>30.7 0.1 -21.0		
P23		11.1 33.9 -13.4<br>11.0 36.3 -10.0<br>11.0 38.6 -6.5<br>10.9 40.9 -3.1	10.6 50.3 10.6 rP23
P24		13.7 35.3 -13.0<br>13.9 36.4 -9.3<br>14.1 37.4 -5.6	14.4 39.5 1.8<br>14.6 40.6 5.5<br>14.8 41. 9.3
P25	24.7 0.3 -23.8<br>28.1 0.4 -23.4<br>31.5 0.6 -23.1	4.9 42.0 -14.5<br>5.8 42.7 -10.8	14.6 40.0 1.0<br>14.8 41.0 4.7<br>15.0 42.0 8.5
P26	19.8 -3.8 -15.9<br>23.4 -3.9 -15.9<br>27.0 -3.9 -15.9<br>30.7 -3.9 -15.9	4.9 42.0 -14.5<br>5.8 42.7 -10.8	6.7 39.2 -4.8<br>7.3 39.9 -0.7
P28		8.8 32.7 -12.4	10.9 37.0 2.5<br>11.4 38.1 6.2<br>11.9 39.2 10.0
P33	20.0 -3.9 -16.1<br>23.6 -3.8 -15.7<br>27.1 -3.7 -15.4<br>30.7 -3.6 -15.0		
P34	24.8 -8.5 -11.5<br>28.2 -8.6 -11.7		

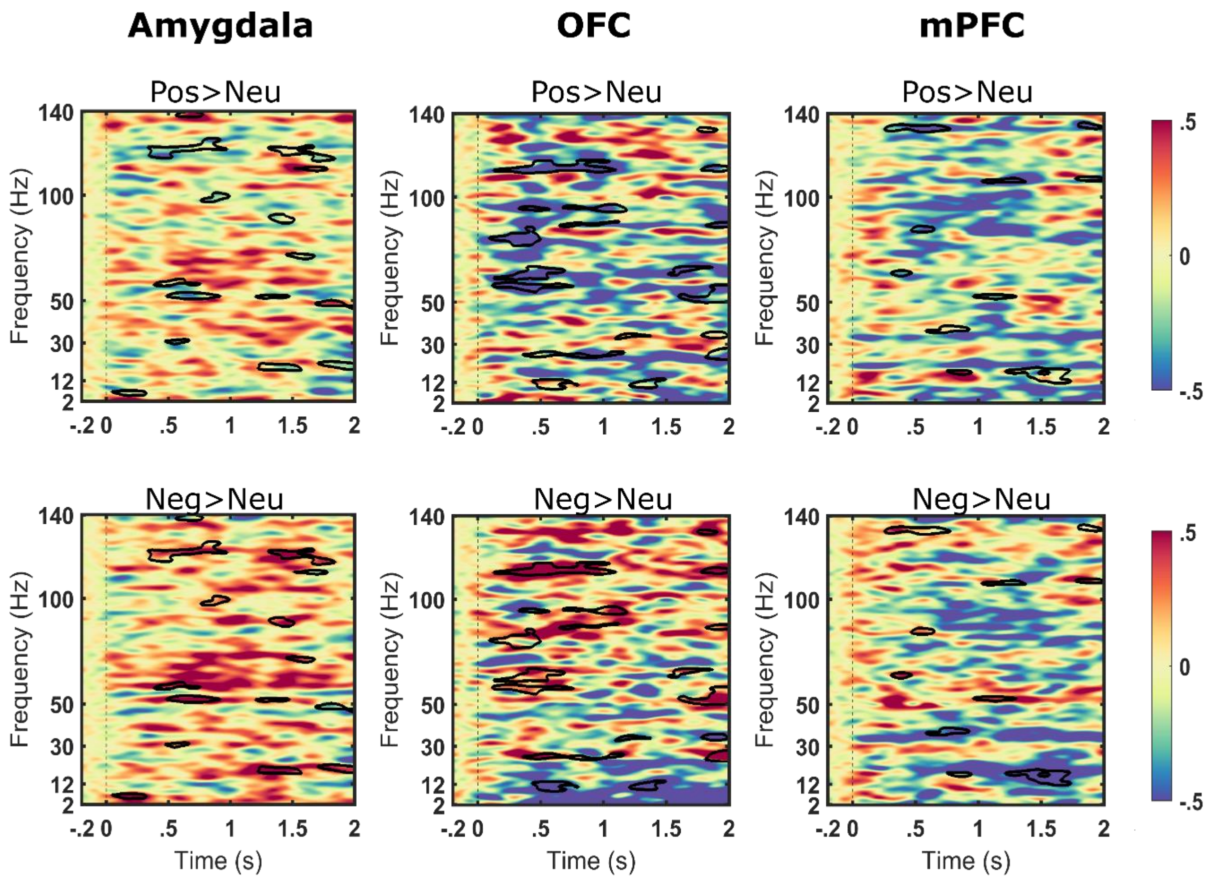

SFigure 1. Task induced activity relative to neutral condition. Grand averaged event related spectral perturbation (ERSP) maps for the amygdala, the orbito-frontal cortex (OFC) and medial prefrontal cortex (mPFC) for positive (Pos - top), negative (Neg - middle) and neutral (Neu - bottom) conditions. Warmer colours denote task-induced power increases from the baseline, while cooler colours refer to power decreases from the baseline. Significant clusters from the statistical permutation testing shown in black outline (see methods). Size of clusters for significance testing was thresholded at 500 and above.

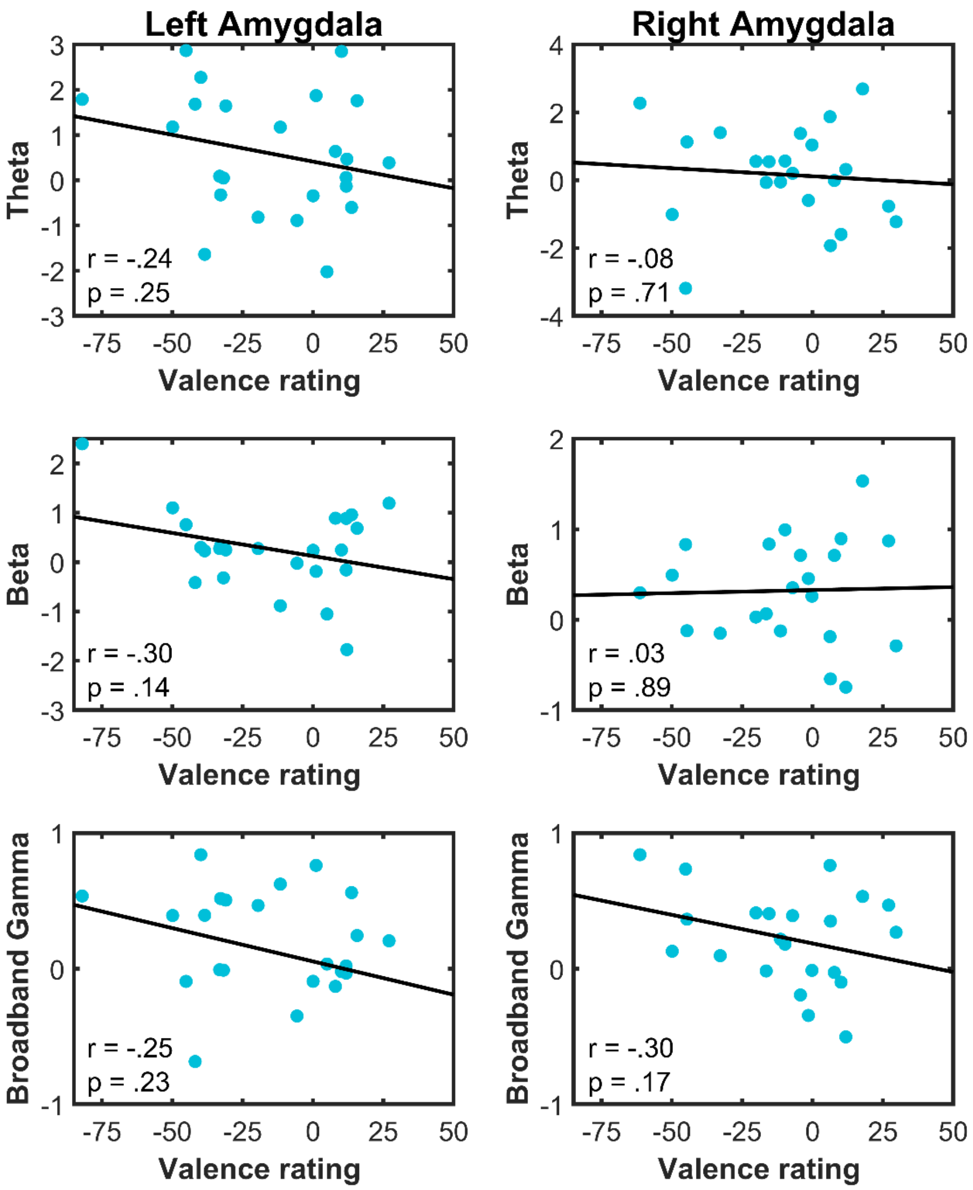

SFigure 2. Association of left and right amygdala activity with valence ratings. Left – association of valence ratings (relative to neutral) with left amygdala activity; theta (top), beta (middle) and broadband gamma (bottom). Right – association of valence ratings (relative to neutral) with left amygdala activity; theta (top), beta (middle) and broadband gamma (bottom). No significant associations were found. Multiple comparison applied for overall 6 tests $p_{FDR}<.05$. Unadjusted p-values are shown.

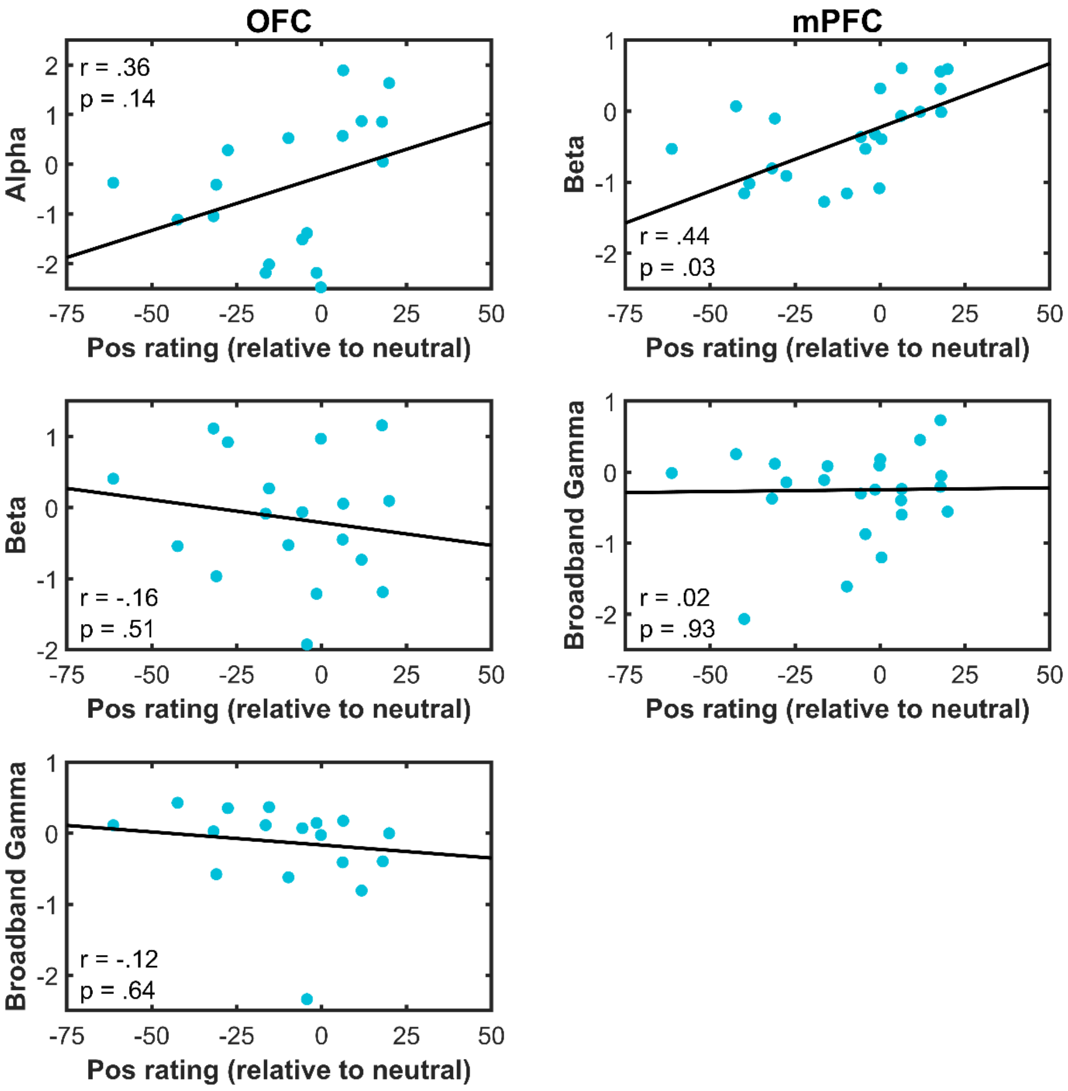

SFigure 3. Association of orbitofrontal cortex (OFC) and medial prefrontal cortex (mPFC) activity with valence ratings. Left – no significant association of OFC activity with valence ratings (relative to neutral): alpha activity (top), beta activity (middle) and broadband gamma (bottom). Right – no significant association of mPFC activity with valence ratings (relative to neutral): beta activity (top) and broadband gamma (bottom). Multiple comparisons applied for 5 comparisons at $p_{FDR}<.05$. Unadjusted p-values are shown. Unadjusted p-values are shown.

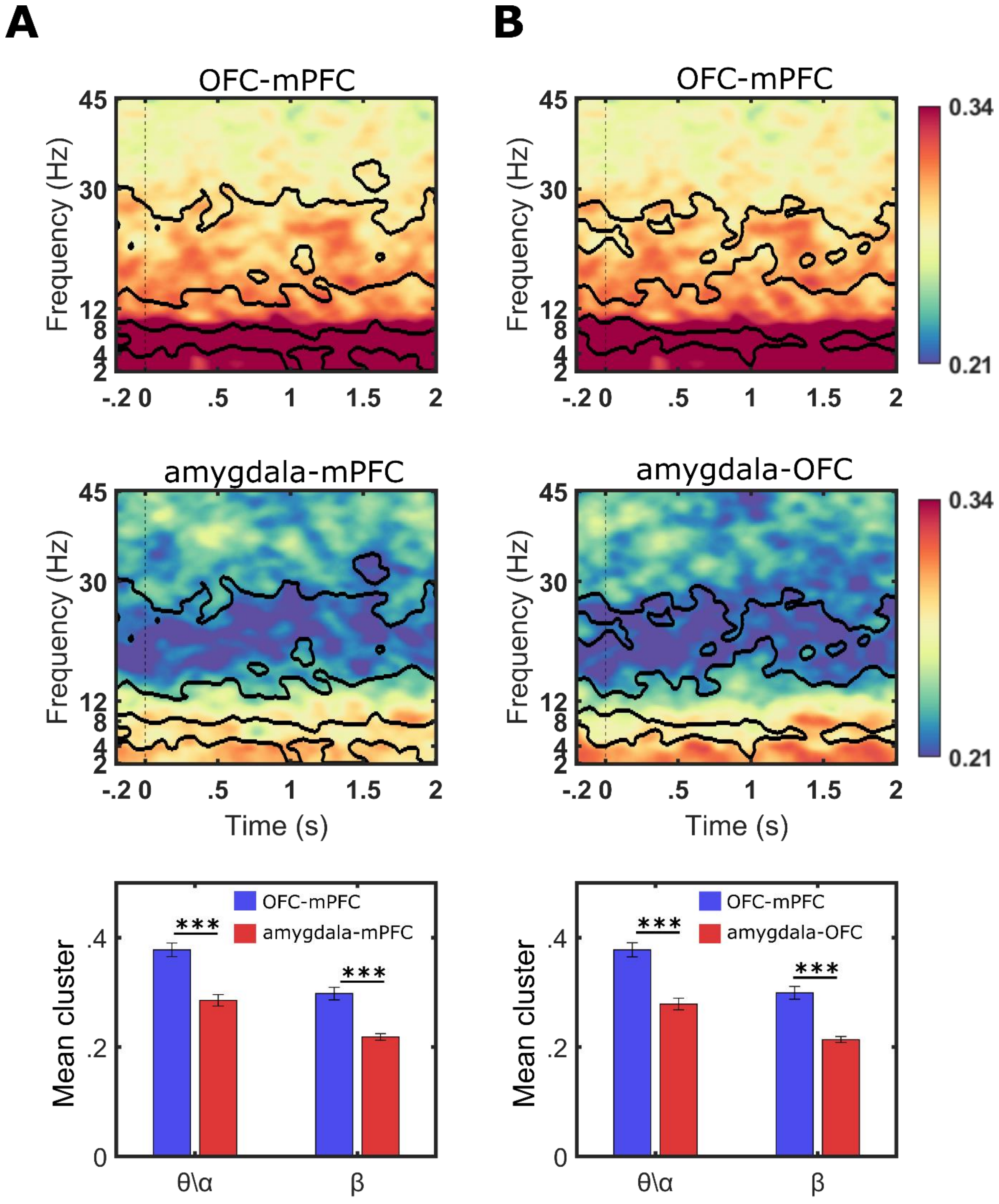

SFigure 4. Coherence differences between the dyads. A. Grand averaged (all conditions) coherence maps for the orbitofrontal cortex (OFC)-medial prefrontal cortex (mPFC) (top) and amygdala-mPFC (middle) with mean of the clusters shown in the bar plots (bottom). Theta/ Alpha (OFC-mPFC: .38 (.10); amygdala-mPFC: .28 (.01); $t_{248} = 6.57$, $P_{FDR} = 1e^{-10}$), beta (OFC-mPFC: .30 (.01); amygdala-mPFC: .22 (.006); $t_{248} = 5.98$, $P_{FDR} = 4e^{-10}$). **B.** Grand averaged (all conditions) coherence maps for the OFC- mPFC (top) and amygdala-OFC (middle) with mean of the clusters shown in the bar plots (bottom). Warmer colours denote higher coherence, while cooler colours refer to lower coherence values. Significant differences (p<.0001) between conditions shown in black outlines. Bar plots show the group mean of the significant frequency clusters on post-hoc t-tests after multiple comparison corrections. Errors bars indicate standard error. ***p_{FDR}<.001). Theta/Alpha (OFC-mPFC: .37 (.01); amygdala-OFC: .27

(.01); $t_{230} = 4.55$, $P_{FDR} = 8e^{-8}$), beta (OFC-mPFC: .30 (.01); amygdala-OFC: .22 (.006); $t_{230} = 6.23$, $P_{FDR} = 3e^{-9}$).

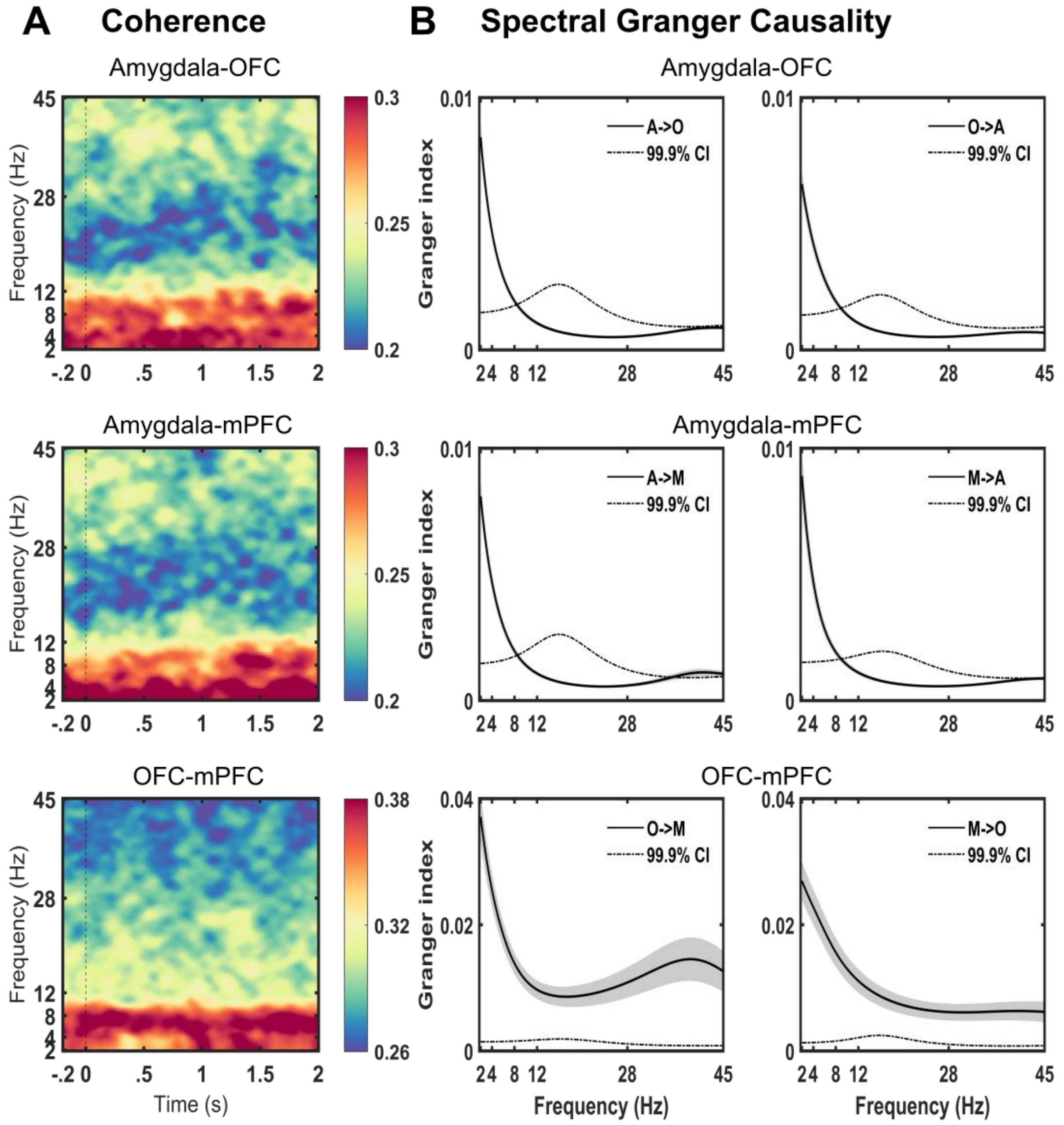

SFigure 5. Functional connectivity. A. Grand averaged time varying coherence plots (averaging over all conditions) highlights strong connectivity in the lower frequency range of <12 Hz. **B.** Spectral granger causality (sGC) results. Top: Amygdala (A)-orbitofrontal cortex (O), middle: Amygdala (A) - medial prefrontal cortex (M), bottom: OFC (O)-mPFC (M). Dotted lines show 99.9 % confidence interval (CI) after 1000 permutation testing. sGC analyses confirm the results from coherence analysis suggesting predominant connectivity in the low frequency range.

DCM analyses on 3-node network

We undertook DCM analyses from a subset of patients with concomitant contacts in all the three regions. For a 2-node network, it is possible to investigate all possible connectivity models as the model space is limited. On the other hand, model space for 3 or more node network is extremely large and leads to issues of "combinatorial explosion" (Stephan, Penny et al. 2010, Whittaker 2013) limiting an exhaustive testing of a full model space. Therefore, defining a relevant model space, given relevant priors can be used to constrain the model space (Stephan, Penny et al. 2010, Friston 2011). Previous work has demonstrated reducing the model space by partitioning the 3-node network into 3 two-node sub-systems (Whittaker 2013) thus vastly reducing the model space needed for an exhaustive search thereby saving computation time from weeks to days.

Adopting similar strategy, we built our model space for 3-node network, from the models with maximum exceedance probability >.1% from 2 node DCM results (reported in the main manuscript Figure 5). These combinations thus led to a model space with 12 models in a 3-node network (supplementary figure 6 top). For computational efficiency we also downsampled the data to 200 Hz. Bayesian model selection identified model 2 as the winning model (69%) (supplementary figure 6 bottom) which comprised of unidirectional connection from mPFC to amygdala, amygdala to OFC and bidirectional connectivity between OFC and mPFC. Although the mPFC to amygdala connectivity was consistent with our dyadic DCM results, one differing characteristic of the winning 3 node model was the amygdala to OFC influence. It is possible that different and limited subjects may have yielded differing results which, however, warrants further validation in bigger sample sized studies.

Model Space

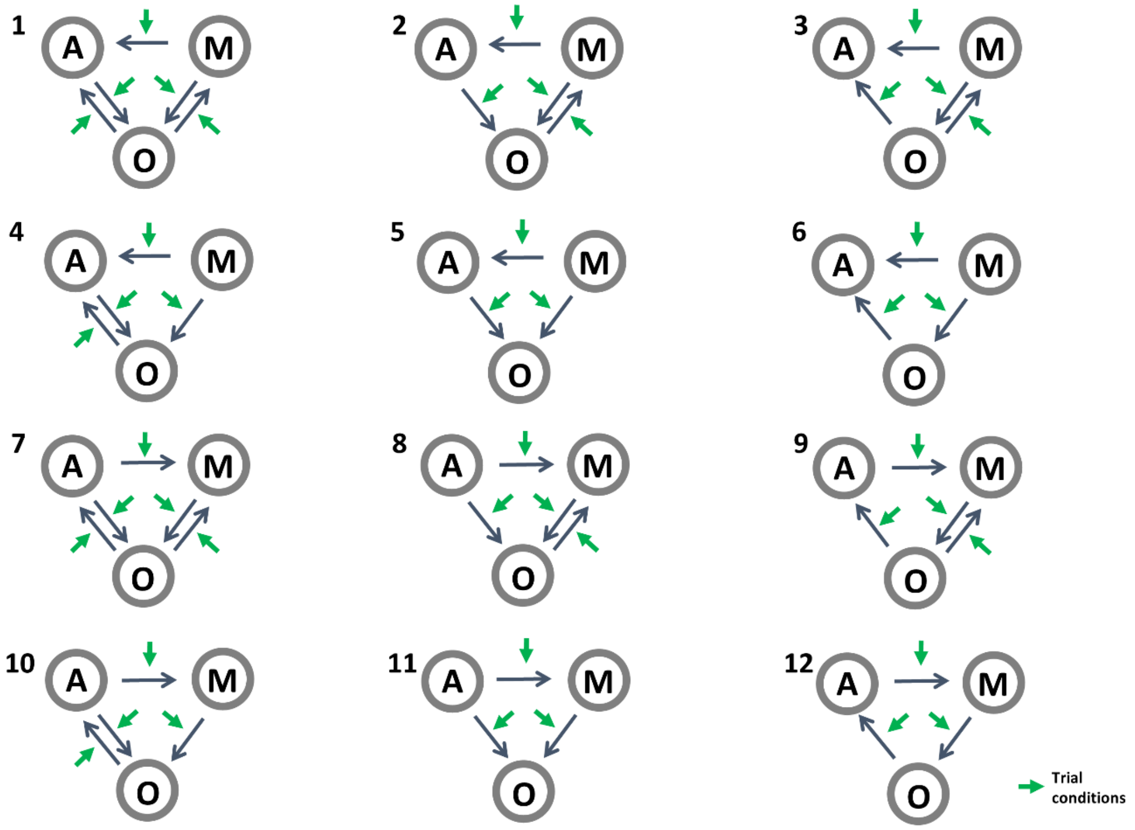

Bayesian Model Selection

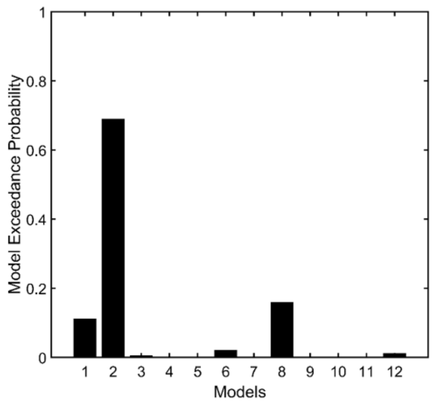

SFigure 6. Dynamic causal modelling (DCM) (2-45Hz) on 3-node network from subset of participants with concomitant contacts in amygdala, orbitofrontal cortex (OFC) and medial prefrontal cortex (mPFC). A. The model space comprised twelve models. The models comprised of connections incorporated from the the winning models with >.1 probability in the 2 node effective connectviity results shown in Figure 5. **B.** Bayesian model selection identified model 2 as the winning model (69%) which comprised of unidirectional connection from mPFC to amygdala, amygdala to OFC and bidirectional connectivity between OFC and mPFC. DCM results might be subject to model space tested and with three nodes here, an exhaustive model space exploration was not undertaken but rather motivated by our prior results.

References

Friston, K. (2011). "Dynamic causal modeling and Granger causality Comments on: The identification of interacting networks in the brain using fMRI: Model selection, causality and deconvolution." Neuroimage **58**(2-2): 303.

Stephan, K. E., W. D. Penny, R. J. Moran, H. E. den Ouden, J. Daunizeau and K. J. Friston (2010). "Ten simple rules for dynamic causal modeling." Neuroimage **49**(4): 3099-3109.

Whittaker, J. (2013). A Method of Reducing Model Space for Dynamic Causal Modelling, University of Manchester.
